# Angiotensin II Removes Kidney Resistance Conferred by Ischemic Preconditioning

**DOI:** 10.1155/2014/602149

**Published:** 2014-08-28

**Authors:** Hee-Seong Jang, Jee In Kim, Jinu Kim, Jeen-Woo Park, Kwon Moo Park

**Affiliations:** ^1^Department of Anatomy and BK21 Plus Biomedical Convergence Program, Cardiovascular Research Institute, Kyungpook National University School of Medicine, 101 Dongin-dong Jung-gu, Daegu 700-422, Republic of Korea; ^2^Department of Molecular Medicine and ODR Medical Research Center, Keimyung University School of Medicine, Daegu 704-701, Republic of Korea; ^3^Department of Anatomy, Jeju National University School of Medicine, Jeju 690-756, Republic of Korea; ^4^School of Life Sciences and Biotechnology, College of Natural Sciences, Kyungpook National University, Daegu 702-701, Republic of Korea

## Abstract

Ischemic preconditioning (IPC) by ischemia/reperfusion (I/R) renders resistance to the kidney. Strong IPC triggers kidney fibrosis, which is involved in angiotensin II (AngII) and its type 1 receptor (AT1R) signaling. Here, we investigated the role of AngII/AT1R signal pathway in the resistance of IPC kidneys to subsequent I/R injury. IPC of kidneys was generated by 30 minutes of bilateral renal ischemia and 8 days of reperfusion. Sham-operation was performed to generate control (non-IPC) mice. To examine the roles of AngII and AT1R in IPC kidneys to subsequent I/R, IPC kidneys were subjected to either 30 minutes of bilateral kidney ischemia or sham-operation following treatment with AngII, losartan (AT1R blocker), or AngII plus losartan. IPC kidneys showed fibrotic changes, decreased AngII, and increased AT1R expression. I/R dramatically increased plasma creatinine concentrations in non-IPC mice, but not in IPC mice. AngII treatment in IPC mice resulted in enhanced morphological damage, oxidative stress, and inflammatory responses, with functional impairment, whereas losartan treatment reversed these effects. However, AngII treatment in non-IPC mice did not change I/R-induced injury. AngII abolished the resistance of IPC kidneys to subsequent I/R via the enhancement of oxidative stress and inflammatory responses, suggesting that the AngII/AT1R signaling pathway is associated with outcome in injury-experienced kidney.

## 1. Introduction

When organs experience injury, such as ischemia/reperfusion (I/R) injury, they become resistant to future insults. This phenomenon, termed as ischemic preconditioning (IPC), has been investigated in order to develop pharmacological agents and surgical procedures to reduce injury, which is inevitable in various clinical settings, such as transplantation and cardiac bypass surgery [1, 2]. There are two classes of IPC, acute/early IPC and late/delayed IPC. IPC is categorized, based on the time between I/R injuries following the brief initial I/R, which does no functional damage in organs [3]. In previous studies we established an animal model of ischemic preconditioning, in which the protective effect of previous I/R injury is dependent on the severity of the previous I/R insult [4–6]. In these studies, the protective effect of ischemic preconditioning, using 30 minutes of ischemia and 8 days of reperfusion, was very strong and almost permanent [6–8]. However, although kidneys that are exposed to strong IPC functionally recover, tubular damage and the expansion of fibrotic lesions, including increases of extracellular matrix level, interstitial cell numbers, and interstitial area, remain [9–12]. This suggests that fibrotic changes and the production of fibrosis-related factors by IPC are associated with the resistance of kidneys to subsequent injury.

The renin-angiotensin system plays a critical role in I/R injury and the progression of fibrosis in kidneys. It has been reported that losartan, a blocker of the angiotensin II (AngII) type 1 receptor (AT1R), inhibits the plasma creatinine increases observed following I/R insult [13, 14]. AngII/AT1R signaling is tightly linked to the progression of kidney fibrosis in chronic kidney diseases [15, 16]. Accordingly, AngII and its receptors appear to be strongly associated with I/R injury and the progression of kidney fibrosis [13–16]. The effects of the AngII/AT1R signaling pathway are known to be associated with oxidative stress, which is a recognized mediator of I/R-induced cell damage and fibrosis progression [9, 17, 18]. AngII induces oxidative damage by producing excessive amounts of reactive oxygen species (ROS) [15, 17]. Furthermore, it has been reported that fibrotic progression and the injury-experienced kidneys are susceptible to chronic administration of AngII through enhanced vascular resistance [12], suggesting that AngII and its receptor enhance the responsiveness of IPC kidneys to injury. However, the role of AngII and its receptor in the susceptibility of IPC kidneys to subsequent I/R insult remains unclear. Therefore, we investigated whether AngII/AT1R is associated with changes in kidney responsiveness due to fibrotic changes from previous I/R injury.

## 2. Materials and Methods

### 2.1. Animal Preparation

All experiments were conducted on 8-week-old male C57BL/6 mice and were approved by the Institutional Animal Care and Use Committee of Kyungpook National University. Mice were allowed free access to water and standard mouse chow. Animals were anesthetized with pentobarbital sodium (60 mg/kg body wt i.p) (Sigma, St. Louis, MO). Kidney ischemia was induced as previously described [4]. In brief, ischemia was induced by clamping both renal pedicles with nontraumatic microaneurysm clamps (Roboz Surgical Instruments, Washington, DC). Incisions were temporarily closed during the ischemia. After clamps were removed, reperfusion was confirmed visually. During operation, body temperature was maintained at 36.5–37.5°C. To generate IPC, mice were subjected to 30 minutes of bilateral renal ischemia and 8 days of reperfusion [4, 7, 8, 19]. To investigate the contributions of angiotensin II (AngII) and type 1 AngII receptor (AT1R) to IPC kidney resistance, some pre-I/R mice were treated subcutaneously with AngII (3 mg/kg body wt, Sigma) [20], losartan (a blocker of AT1R, 25 mg/kg body wt, USD) [13], AngII plus losartan, or 0.9% saline (vehicle) 10 minutes before subsequent 30 minutes of bilateral renal ischemia or sham-operation.

After euthanasia kidneys were either snap-frozen in liquid nitrogen for biochemical experiments or perfusion-fixed in PLP (4% paraformaldehyde, 75 mM L-lysine, and 10 mM sodium periodate; Sigma) for histological studies. To prepare kidney sections, PLP-fixed kidneys were washed with phosphate-buffered saline (PBS) three times for 5 minutes, embedded in paraffin, and cut into 2 *μ*m section with a microtome (RM2165; Leica).

### 2.2. Western Blot Analysis

Western blot analyses were conducted as previously described [4] using anti-AT1R (Santa Cruz, 1 : 1000), -AngII (Novus, 1 : 1000), -Nox2 (BD transduction, 1 : 5000), -Nox4 (Santa Cruz, 1 : 5000), -nitrotyrosine (Cayman, 1 : 1000), -Ly6G (ebioscience, 1 : 1000), and -GAPDH (Santa Cruz, 1 : 5000) antibodies. Densities of the blots were quantified using the ImageJ program (NIH, Bethesda, MD).

### 2.3. Immunohistochemistry

To detect AngII and AT1R, immunohistochemical staining was performed as described previously [11]. AngII (1 : 100; Novus), AT1R (1 : 50; Santa Cruz), and F4/80 (1 : 100; AbD Serotec) antibodies were used. Sections were observed under a LeicaDM2500 (Leica) microscope. Pictures were taken in the outer medulla.

### 2.4. Scoring of Tubular Damage

PLP-fixed kidneys were embedded in paraffin and cut into 2 *μ*m sections, which were stained with periodic acid Schiff (PAS) using a standard protocol. As described previously [7], four kidneys were analyzed per experimental animal group to determine the severity of the tubular cell damage.

### 2.5. Measurement of Blood Pressure

Blood pressure (BP) was evaluated using a noninvasive tail-cuff system (CODA 2, Kent Scientific Corp., Torrington, CT) [21–23].

### 2.6. Measurement of Lipid Peroxidation

As described previously [24], excised kidneys were immediately homogenized in a sucrose buffer (0.32 M sucrose, 10 mM Tris-HCl, and pH 7.4; Sigma) on ice using a Dounce homogenizer. Lipid peroxidation in the samples was evaluated by measuring malondialdehyde (MDA) levels using a spectrophotometric assay for thiobarbituric acid-reactive substances.

### 2.7. Statistics

Results are expressed as the mean ± SEM. Statistical differences between groups were determined using Student's *t*-test. Differences between groups were considered statistically significant when *P* values were < 0.05.

## 3. Results

### 3.1. IPC Kidneys Exhibited Fibrotic Lesions

**Figure 1:**
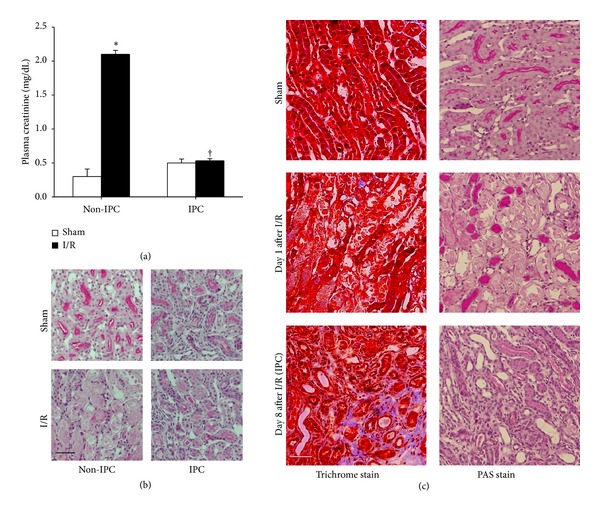
Morphological changes in kidney tissues and increased resistance of IPC kidneys to subsequent I/R insult. Mice were subjected to either 30 minutes of bilateral renal ischemia (IPC) or a sham-operation (non-IPC) on day 0. At 8 days after initial surgery, pre-IPC or non-IPC mice were exposed to either a further 30 minutes of bilateral renal ischemia/reperfusion (I/R) or sham-operation (Sham). 24 hours after second surgery plasma creatinine concentrations (a) and morphological changes (b) were determined. (c) Eight days after I/R, kidneys were harvested and kidney sections were PAS- and Masson's trichrome-stained. In Masson's trichrome-stained tissues areas of collagen deposition appeared in blue color. Images were taken from the outer medulla of kidneys. Scale bar: 50 *μ*m. Values are means ± SEM (*n* = 3–6). *P < 0.05 versus sham in non-IPC; ^†^ *P* < 0.05 versus ischemia in non-IPC mouse.

Thirty minutes of ischemia in non-IPC mice significantly increased plasma creatinine levels (PCr) after 24 hours (Figure 1(a)). The PCr levels in sham-operated IPC mice were not different from sham-operated non-IPC mice, indicating that the increase in PCr due to I/R insult returned to normal after 8 days (Figure 1(a)). Thirty minutes of ischemia in non-IPC mice induced severe morphological damage in the kidneys, as compared to sham-operated non-IPC kidneys. However, morphological damage was not observed in IPC mice after ischemia when compared with sham-operated IPC kidney (Figure 1(b)). Thirty minutes of ischemia resulted in disruption, thinning, dilatation, congestion of tubules, and increases in interstitial cells after 24 hours (Figure 1(c)). At 8 days after I/R, the IPC kidneys were partially recovered and showed atrophied tubules and typical fibrotic lesions, including expansion of interstitium due to increases of interstitial cell numbers and extracellular matrix proteins including collagens (Figure 1(c)). These findings suggest that morphological changes and the factors in IPC kidneys are involved in resistance to subsequent I/R insult.

### 3.2. IPC Kidneys Exhibited Low Levels of AngII and High Levels of AT1R Expression

**Figure 2:**
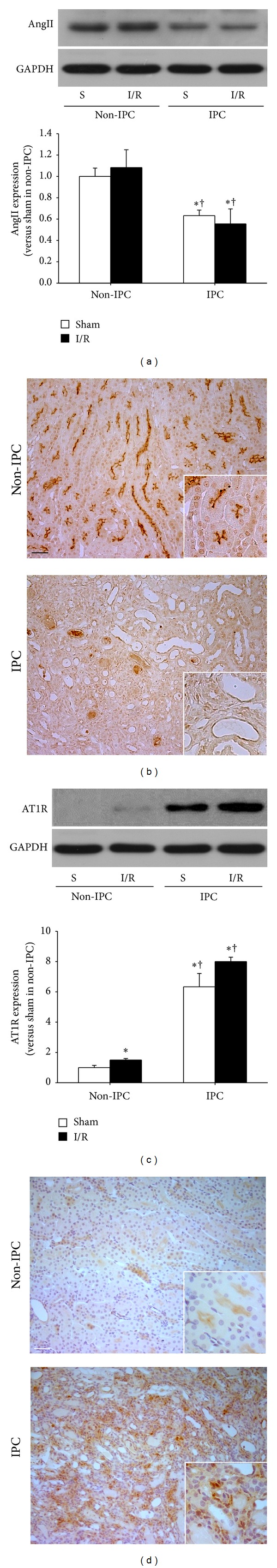
Expression of AngII and AT1R in non-IPC and IPC kidneys. IPC or non-IPC mice were exposed to either 30 minutes of bilateral renal ischemia/reperfusion (I/R) or a sham-operation (Sham). 24 hours after second surgery, kidneys were harvested. (a) AngII and (c) AT1R expression were determined by western blot using anti-AngII antibody. The AngII band is predominant at ~40 kDa. GAPDH expression was used to confirm equal protein loading. The densities of the blots were quantified using the ImageJ program. Serially sectioned paraffin-embedded kidney tissues were immunostained with (b) AngII (brown) and (d) AT1R (brown) antibodies as described in Section 2. Arrows indicate AngII-positive proximal tubules. Hematoxylin was used to detect nuclei (blue). Pictures were taken of the outer medulla in kidneys. S indicates sham. Scale bar: 50 *μ*m. Values are means ± SEM (*n* = 3–5). *P < 0.05 versus sham in non-IPC; ^†^ *P* < 0.05 versus I/R in non-IPC.

To investigate the involvements of AngII and AT1R in IPC kidney resistance, we first determined the expression levels and localization of AngII in kidneys. AngII levels in sham-operated IPC kidney lysates were significantly lower than those of sham- or I/R-induced non-IPC mice (Figure 2(a)), indicating that I/R gradually decreased AngII expression. On the other hand, ischemia in both the non-IPC and IPC mice did not significantly change the AngII expression after 24 hours when compared with their respective sham mice (Figure 2(a)). Further, we examined the localization of AngII in IPC kidneys. AngII was extensively expressed in the brush border of proximal tubules in the outer medulla of non-IPC kidneys. In contrast, only faint expression of AngII was observed in the proximal tubule cells of IPC kidneys, indicating that I/R insult induced the reduction of AngII in the proximal tubules (Figure 2(b)). These findings suggest that changes in the expression level and localization of AngII may be associated with the ability of IPC kidneys to tolerate subsequent I/R insults.

Next, we determined the expression levels of AT1R in the kidneys. AT1R levels in both sham-operated and I/R-induced IPC kidney lysates were significantly greater than those of sham- or I/R-induced non-IPC mice (Figure 2(c)). However, after 30 minutes of ischemia in non-IPC mice, there was a significant but mild increase in AT1R expression after 24 hours, as compared with non-IPC sham mice. However, this effect was not observed in IPC mice (Figure 2(c)). In non-IPC kidneys, AT1R signal was mainly observed in the tubular epithelial cells, but the AT1R signal was very weak. In IPC-kidneys AT1R signal was mainly observed in the interstitial cells and the AT1R signal was very strong (Figure 2(d)). Therefore, the increase of AT1R in the IPC-kidney lysates may be associated with the increased interstitial cells, including fibroblasts and accumulated inflammatory cells. Many portions of those interstitial cells may reflect an increased number of macrophages, which are major players in the progression of fibrosis and express AT1R [25]. These findings suggest that changes in the expression level and localization of AngII and AT1R are associated with the ability of IPC kidneys to tolerate subsequent I/R insults.

### 3.3. AngII Administration Removed the Tolerance of IPC Kidneys to I/R Insult

**Figure 3:**
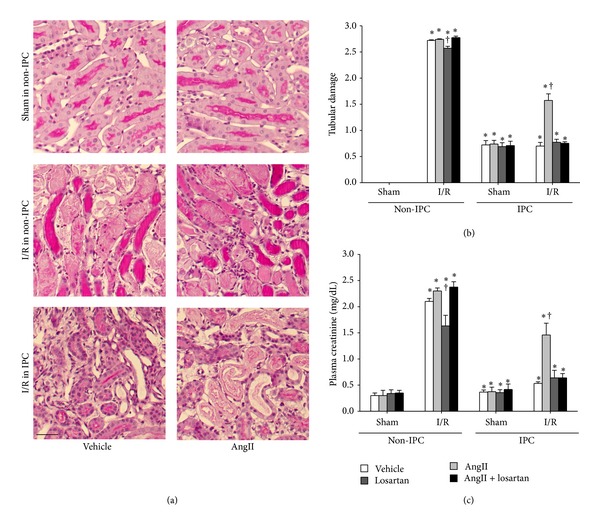
Plasma creatinine concentrations 24 hours after I/R in non-IPC and IPC mice. IPC or non-IPC mice were exposed to either 30 minutes of bilateral renal ischemia/reperfusion (I/R) or a sham-operation (Sham). At 10 minutes before the second surgery, mice were treated with AngII (3 mg/kg body weight, BW), losartan (25 mg/kg BW), AngII plus losartan (AngII + losartan), or vehicle. (a) 24 hours after second surgery, kidney sections were PAS-stained. Images were taken of the outer medulla of the kidneys. (b) Ten fields per kidney were randomly selected to evaluate tubular damage. (c) To determine plasma creatinine concentrations, plasma was collected at 24 hours after the second surgery. Scale bar: 50 *μ*m. Values are means ± SEM (*n* = 3–6). *P < 0.05 versus vehicle-treated sham in non-IPC; ^†^ *P* < 0.05 versus vehicle-treated ischemia in non-IPC or IPC.

To investigate the roles of AngII and AT1R in the susceptibility of IPC kidneys to I/R insult, we administered AngII to IPC mice 10 minutes before inducing ischemia. AngII treatment in non-IPC mice did not significantly enhance tubular damage observed after I/R insult as compared with vehicle-treated non-IPC mice (Figures 3(a) and 3(b)). In contrast, AngII treatment in IPC mice significantly increased tubular damage scores 24 hours after ischemia as compared with sham-operated controls, whereas vehicle-treatment did not (Figures 3(a) and 3(b)). In line with tubular damage scoring results, AngII administration increased PCr concentrations following subsequent I/R insult in IPC mice. However, in non-IPC mice, AngII increased functional loss in the kidney, although not significantly when compared with vehicle-treated controls (Figure 3(c)). Indeed, approximately 60% of functional resistance in IPC kidneys was lost following AngII treatment (Figure 3(c)).

Treating non-IPC mice with losartan, a blocker of AT1R, significantly inhibited the increase in PCr observed after ischemia. However, simultaneous treatment with AngII and losartan did not affect change of postischemic PCr levels as compared with vehicle treatment (Figure 3(c)). In contrast, losartan alone did not affect the postischemic PCr levels in IPC mice as compared with vehicle-treatment. However, the administration of AngII with losartan in IPC mice prevented I/R-induced PCr increases caused by AngII (Figure 3(c)). Tubular damage scores concurred with PCr levels in the non-IPC and IPC kidneys (Figures 3(a) and 3(b)). These results indicate that the AngII/AT1R pathway is associated with the resistance of IPC kidneys against subsequent I/R insult.

### 3.4. AngII Enhanced Oxidative Stress and Inflammatory Responses after I/R in IPC Kidneys

**Figure 4:**
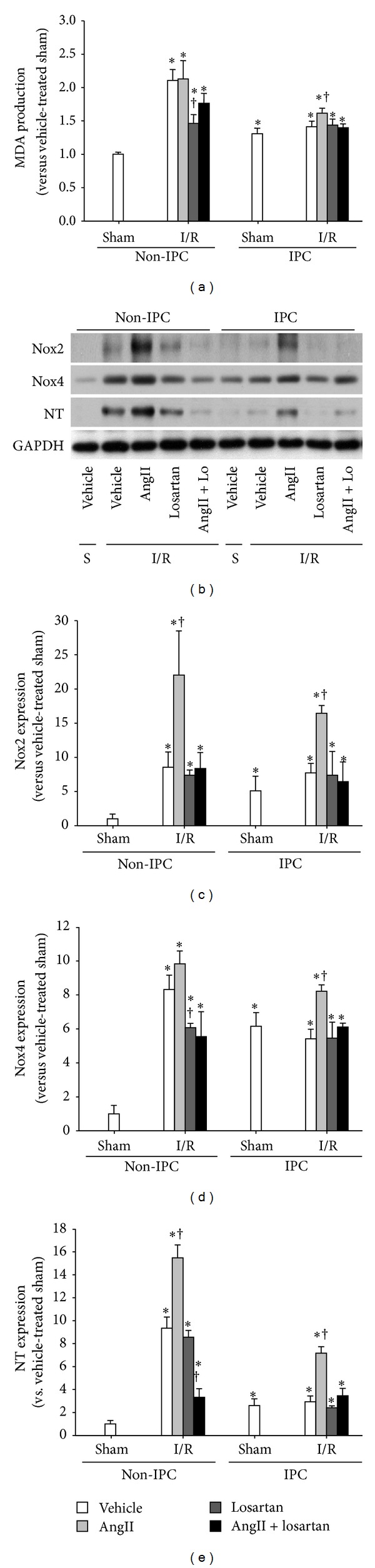
Levels of oxidative stress after I/R in non-IPC and IPC kidneys. IPC or non-IPC mice were exposed to 30 minutes of bilateral renal ischemia/reperfusion (I/R) or a sham-operation (Sham). Ten minutes before second surgery, mice were treated with AngII (3 mg/kg body weight, BW), losartan (Lo, 25 mg/kg BW), AngII plus losartan (AngII + Lo), or vehicle. 24 hours after second surgery, lipid peroxidation was evaluated by assessing (a) MDA levels and (b) Nox2, Nox4, and nitrotyrosine (NT) expression by western blot. GAPDH expression was used to confirm equal protein loading. ((c)–(e)) The densities of the blots were quantified using the ImageJ program. S indicates sham. Values are means ± SEM (*n* = 3–5). *P < 0.05 versus sham in non-IPC; ^†^ *P* < 0.05 versus vehicle-treated ischemia in non-IPC or IPC.

Since the AngII/AT1R signal pathway is associated with oxidative stress and inflammatory responses, which are a major factor in I/R injury [26, 27], we determined whether the effect of the AngII/AT1R signal pathway in IPC kidneys is associated with oxidative stress and inflammatory responses. Postischemic increases in malondialdehyde (MDA) production in IPC kidney were significantly lower than in non-IPC kidneys (Figure 4(a)). AngII administration in IPC mice increased the postischemic MDA level (Figure 4(a)), and this enhancement was prevented by simultaneous treatment of losartan (Figure 4(a)). In non-IPC mice, losartan treatment reduced postischemic increases in MDA, and the effect of losartan was mitigated by simultaneous administration of AngII (Figure 4(a)). Furthermore, the expression of Nox2, Nox4, and nitrotyrosine changed in a manner similar to MDA level (Figures 4(b)–4(e)). Nitrotyrosine is a product of peroxynitrite, a potent oxidant that produces nitric oxide-derived oxidants, including nitrotyrosine by promoting the nitration of protein tyrosine residues [28, 29]. These data indicate that the changes in the tolerance of kidneys to I/R insult in IPC kidneys induced by AngII are associated with oxidative stress.

**Figure 5:**
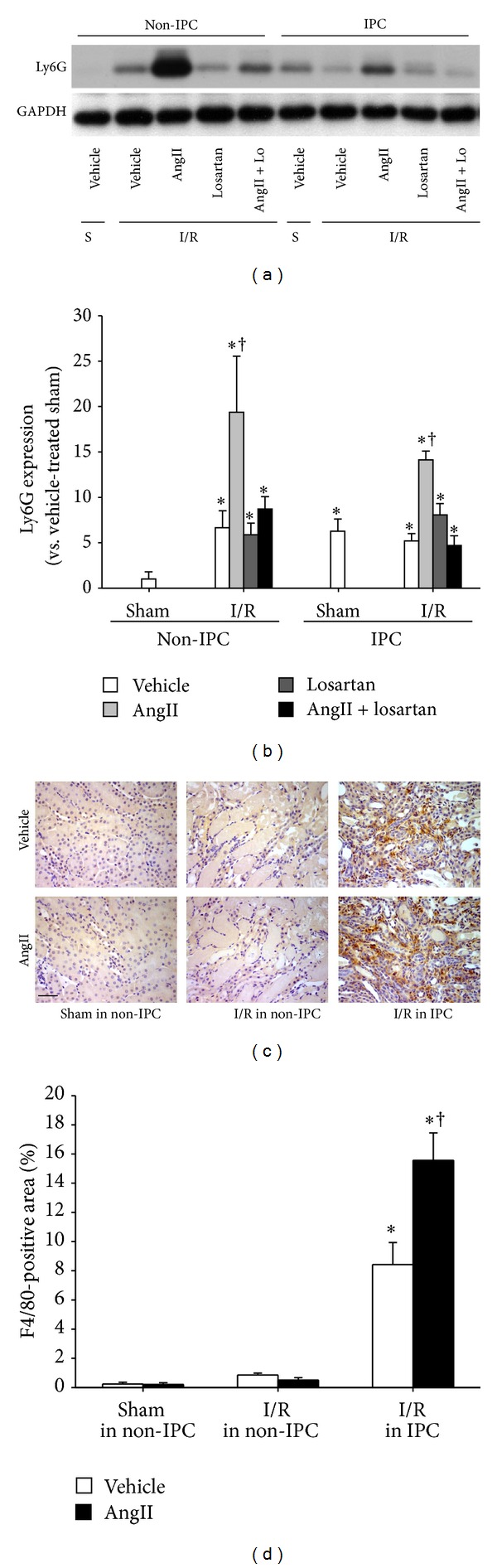
Levels of inflammation after I/R in non-IPC and IPC kidneys. IPC or non-IPC mice were exposed to 30 minutes of bilateral renal ischemia/reperfusion (I/R) or a sham-operation (Sham). Ten minutes before the second surgery, mice were treated with AngII (3 mg/kg body weight, BW), losartan (Lo, 25 mg/kg BW), AngII plus losartan (AngII + Lo), or vehicle. 24 hours after second surgery, (a) Ly6G expression was determined by western blotting. GAPDH expression was used to confirm equal protein loading. (b) The densities of the blots were quantified using the ImageJ program. (c) Paraffin-embedded kidney sections were immunostained with anti-F4/80 (brown) antibody as described in Section 2. Hematoxylin was used to detect nuclei (blue). Pictures were taken of the outer medulla in kidneys. (d) F4/80-positive areas were measured by using i-Solution software. Scale bar: 50 *μ*m. S indicates sham. Values are means ± SEM (*n* = 3–5). *P < 0.05 versus sham non-pre-IRI; ^†^ *P* < 0.05 versus vehicle-treated ischemia in non-IPC or IPC.

The expression of Ly6G, a marker of neutrophil, and F4/80, a marker of macrophage, was also enhanced in AngII-treated IPC kidneys after subsequent I/R insult, as compared to those of vehicle. The changes induced by AngII in IPC kidneys were reversed by losartan (Figure 5). In the non-IPC kidneys, AngII markedly increased the expression of Ly6G following subsequent I/R insults, as compared with other groups, and did not change the expression of F4/80 (Figure 5). These data indicate that the enhancement of inflammatory response induced by AngII is associated with alterations in kidney tolerance to subsequent I/R insult.

Finally, to examine the involvement of BP in the susceptibility of IPC kidneys to I/R insult, we measured the BP in the mice using a noninvasive tail-cuff method. BP was not significantly different 24 hours after I/R insult. There was no significant difference in BP between all experimental groups (data not shown). This data indicate that BP likely does not play a critical role in the susceptibility of IPC kidneys to subsequent I/R insult.

## 4. Discussion

In the present study, we found that IPC kidneys with fibrotic lesions are less susceptible to I/R insult. Additionally, IPC decreased the expression level of AngII, whereas it increased the expression of AT1R. Alterations in AngII and AT1R localization were also observed. Furthermore, the administration of AngII reduced the resistance of IPC kidneys to subsequent I/R insult, independent of BP. In contrast, the AT1R antagonist losartan completely blocked the effect of AngII in IPC kidneys. Finally, AngII administration enhanced oxidative stress and inflammatory responses following I/R insult in IPC kidneys. Treatment with losartan blocked this effect. Combined, these results indicate that the AngII/AT1R signal pathway is associated with the resistance of IPC kidneys to subsequent I/R insult.

It is well known that organs with a history of injury can better resist subsequent injuries [1–3]. We have previously demonstrated that I/R injury to the kidney confers resistance in an I/R-injury severity-dependent manner [4, 5]. Although more severe I/R insults induce a strong protective effect, it can also cause fibrotic changes in the kidneys. This suggests that fibrosis and fibrosis-related factors are associated with reduction of kidney susceptibility to injury. In the present study, we found that I/R insults gradually decreased AngII levels and changed its localization. In contrast, we found that I/R insult gradually increased AT1R levels and increased its interstitial expression. In the present study, the prominent band of western blot for AngII was detected at ~40 kDa size. It is known that AngII antibody used in this study detects approximately 40 kDa size. However, since AngII peptide is only 8 amino acids, the AngII antibody may cross-react with precursors of AngII. Kontogiannis and Burns reported that proximal tubular AT1R mRNA expression was decreased early after reperfusion followed by ischemia, returning to sham levels by 72 hours [13]. It indicates that the resistance of IPC kidneys to I/R is associated with the change of expression level and localization of AngII/AT1R. In present study, we did not observe significant changes in systemic BP; however, the levels of AngII and AT1R in the kidney significantly changed. It suggests that the local changes of AngII and AT1R in the kidney after I/R injury may not be a major factor in systemic BP regulation and that the balance of AngII and AT1R in the kidney is not important in the regulation of systemic BP. Further studies are required to define exact molecular mechanism between the local changes in kidney of AngII and AT1R and systemic BP.

Recent epidemiological data have suggested that chronic kidney disease (CKD) is a risk factor for the incidence of acute kidney injury (AKI) [30, 31]. However, the underlying mechanism is unknown. In the present study, IPC kidneys recovered from AKI had fibrotic lesions, a common feature in CKD [9], and resisted subsequent I/R insult. AngII abolished the resistance of IPC kidneys to subsequent I/R insults but did not affect non-IPC kidneys, suggesting that the enhanced susceptibility of IPC kidneys to AngII is associated with increased AngII/AT1R signaling. Basile et al. reported that kidneys recovering from I/R injury had enhanced vascular reactivity to AngII [12, 32, 33]. Therefore, the high incidence of AKI in patients with CKD [30, 31] may be associated with the enhancement of AngII/AT1R signaling. Combined, the data suggests that AT1R blockers or ACE inhibitors would be advisable to use for the prevention of AKI in the patients with prospective risk of I/R injury.

Inflammation is a critical factor in the induction of AKI [10]. In our current study, I/R resulted in the increase of Ly6G expression in non-IPC kidneys. However, in IPC mice, subsequent I/R injury did not increase Ly6G expression in the kidney of vehicle-treated mice but enhanced it in AngII-administered mice, suggesting that AngII distinctly regulates leukocyte recruitment into the sites of injury in non-IPC and IPC kidneys. Recent data showed that AT1R in macrophages accelerated the progression of kidney injury-induced atherosclerosis in mice by shifting the macrophage phenotype to inflammatory macrophages [25]. In present study, we observed increases in AT1R expression in interstitial cells, which are composed of fibroblasts and macrophages [11, 34]. Therefore, AngII treatment in IPC kidneys may induce the activation or phenotype-change of preexisting macrophages through macrophage AT1R, and this effect may also be associated with the upregulation of oxidative stress.

Several reports suggest that enhanced vascular reactivity to AngII in animals with post-I/R kidney is related with increased oxidative stress [12, 32, 33, 35]. Under normal physiological conditions, ROS formation and scavenging by the antioxidant system are in homeostatic balance [9, 36]. Recent studies have demonstrated that a mild increase in ROS during the recovery phase after I/R induces kidney, heart, and liver tolerance to subsequent injury [19, 37, 38], despite the fact that excessive ROS contributes to the progression of renal diseases [9, 24]. In a previous study, we reported that a small increase in ROS, such as that shown by IPC kidneys, confers resistance to subsequent I/R insult [19]. The IPC fibrotic kidneys in the present study represent a state of increased lipid peroxidation, with elevated NADPH oxidases and nitrotyrosine levels. This suggests that a mild increase in ROS in IPC kidneys confers resistance to subsequent I/R insults. AngII treatment into mice with IPC kidneys resulted in enhanced lipid peroxidation, Noxs, and nitrotyrosine expression, compared with those of vehicle-treated mice. However, the elevation of MDA, Noxs, and nitrotyrosine levels by AngII in IPC kidneys was prevented by losartan. This suggests that the resistance of IPC kidneys to subsequent I/R insults may be mediated by a slight increase in ROS, whereas the increased susceptibility of AngII-treated IPC kidneys may be associated with high ROS levels. Many studies have demonstrated that AngII and AT1R signals accelerate ROS production [15, 17, 26].

In conclusion, although further studies are required to clearly define the underlying molecular mechanisms, our present findings demonstrate that the AngII/AT1R signaling pathways are critical factor in the susceptibility of IPC kidneys to I/R insult, suggesting that AngII and AT1R are possible targets for the prevention of AKI in the patient with chronic kidney disease.
